# Metabolo-Transcriptomic Analysis Reveals the Mechanisms Underlying Seed Dormancy Release in *Polygonatum sibiricum*

**DOI:** 10.3390/ijms27157032

**Published:** 2026-08-05

**Authors:** Xiaoyu Su, Chunming Li, Lei Li, Yaling Yang, Lina Wang, Yiwen Cao, Dandan Lu, Yao Sun, Mengfan Su, Yongliang Yu, Zhengwei Tan, Huizhen Liang

**Affiliations:** 1 Institute of Chinese Herbal Medicines, Henan Academy of Agricultural Sciences, Zhengzhou 450002, China; suxiaoyu@hnagri.org.cn (X.S.); lchm1212@hnagri.org.cn (C.L.); lilei@hnagri.org.cn (L.L.); yangyaling202@163.com (Y.Y.); hnndlina@163.com (L.W.); yiwencao96@163.com (Y.C.); ludandan0710@163.com (D.L.); ruth_3912834@163.com (Y.S.); sufancy1669@163.com (M.S.); yyl790721@126.com (Y.Y.); 2 Provincial Key Laboratory of Conservation and Utilization of Traditional Chinese Medicine Resources, Zhengzhou 450002, China

**Keywords:** *Polygonatum sibiricum*, seed dormancy, multi-omics, plant hormone signaling, starch and sucrose metabolism

## Abstract

The seeds of *Polygonatum sibiricum* exhibit dormancy, which poses a major challenge for its cultivation. To elucidate the regulatory mechanisms underlying dormancy release, we performed integrated transcriptomic and metabolomic analyses on seeds at 0, 5, 10, and 15 d after seed imbibition. Physiological assays revealed progressive declines in abscisic acid (ABA) and starch levels, alongside increases in gibberellin (GA) and soluble sugar contents, reflecting the metabolic changes accompanying the transition from dormancy to germination. Transcriptomic analysis identified 11,520 expressed genes, with 6753, 7775 and 9387 differentially expressed genes (DEGs) at T5, T10, and T15, respectively. KEGG enrichment highlighted starch and sucrose metabolism and plant hormone signal transduction as key pathways. Notably, the GA biosynthesis gene *GA3ox* was markedly upregulated, while the *DELLA* repressor (Isoform0012761) showed sustained downregulation, suggesting relieved GA signaling. In the ABA pathway, *CYP707A* catabolic genes exhibited biphasic expression, and ABA signaling components (*PYL*, *PP2C*, *SnRK2*) showed stage-specific remodeling. A total of 316, 412, and 479 differentially expressed transcription factors were identified across stages, with the GRAS family being the largest. Co-expression network analysis revealed 19 transcription factors integrating starch/sucrose metabolism with ABA and GA signaling, most of which were downregulated, except one C2H2 member showing sustained upregulation. These findings demonstrate that dormancy release in *P. sibiricum* is governed by coordinated hormonal reprogramming, metabolic mobilization, and transcription factor-mediated regulation, providing a theoretical foundation for improving seed germination in this medicinal plant.

## 1. Introduction

*Polygonatum sibiricum* Red. (Asparagales, Liliaceae) is a perennial herb widely distributed across temperate to frigid zones in the Northern Hemisphere [1]. Its rhizome has been used in traditional Chinese medicine for over 2000 years to nourish yin, moisten the lungs, and invigorate the spleen [2]. Together with *P. kingianum* and *P. cyrtonema*, it is one of the three botanical origins of Polygonati Rhizoma recorded in the 2025 edition of the *Pharmacopoeia of the People’s Republic of China* [3]. Modern pharmacology attributes these effects to polysaccharides, flavonoids, and steroidal saponins, which exhibit antioxidant, immunomodulatory, anti-inflammatory, and anti-aging activities [4,5,6]. As a medicine-food homology species, *P. sibiricum* is also rich in proteins, amino acids, and dietary fiber, making it widely used in functional foods and cosmetics [7,8]. This multifaceted value has driven a surge in market demand, placing pressure on wild resources and making artificial cultivation the primary approach to address the resource shortage of *P. sibiricum*.

The artificial cultivation of *P. sibiricum* relies on two primary propagation methods: rhizome propagation and seed propagation. While rhizome propagation is widely employed in practice, it inevitably leads to the deterioration of medicinal quality due to its asexual nature, which limits its long-term sustainability [9]. On the other hand, seed propagation offers advantages for germplasm improvement and innovation, yet it is severely constrained by the intrinsically dormant seeds of *P. sibiricum*. Under natural conditions, *P. sibiricum* seeds require more than one year to complete germination, primarily due to a combination of physical barriers (lignified seed coats impairing water uptake), physiological blocks (elevated ABA content), nutritional constraints (inefficient mobilization of endosperm reserves), and the presence of endogenous inhibitors in seed coats, endosperms, and embryos [10,11,12]. Although various dormancy-breaking treatments, such as cold stratification and exogenous hormone applications, have been explored to address this issue [13,14,15], most efforts have focused on empirical method optimization rather than on elucidating the underlying molecular mechanisms. Nevertheless, the molecular basis of seed germination has been well characterized in other plant species, where it is governed by a complex interplay of phytohormones, transcriptional reprogramming, and metabolic shifts, with the antagonistic ABA/GA balance acting as the central regulatory hub [16,17]. In *P. sibiricum*, however, hormonal profiling has shown that ABA prevails during dormancy, whereas GA_3_ and jasmonic acid surge at the time of seed coat rupture, and IAA accumulates progressively throughout germination [14]. Transcriptomic and metabolomic analyses in the congeneric species *P. cyrtonema* have further pinpointed plant hormone signal transduction, starch and sucrose metabolism, and phenylpropanoid/flavonoid biosynthesis as key pathways associated with germination [18,19]. However, whether these regulatory modules are conserved in *P. sibiricum* remains unknown, highlighting the need for systematic investigation of the early molecular events governing dormancy release in *P. sibiricum*.

Transcription factors (TFs) serve as key nodes that integrate hormonal signals and orchestrate downstream transcriptional reprogramming during seed germination [20]. Numerous TFs have been functionally characterized in model plants: in *Arabidopsis*, *MYB96* and *MYB30* regulate ABA signaling and dormancy breaking [21]; *ABI5* (bZIP) is a central regulator of ABA signaling during germination [22]; and WRKY25/36 modulate dormancy through *DELAY OF GERMINATION 1* (*DOG1*) [23,24]. Beyond *Arabidopsis*, functionally analogous TFs have also been identified in rice, tomato, and maize [25,26,27]. In *P. cyrtonema*, three MYB transcription factors (*PcMYB4*, *PcKUA1*, and *PcCSA*) were shown to promote germination by repressing cytokinin oxidase genes (*PcCKX1,2,3*), and their heterologous expression in *Arabidopsis* repressed dormancy-associated genes such as *DOG1*, *NCED3*, and *ABI5* [28]. In *P. kingianum*, WGCNA identified *DOF AFFECTING GERMINATION 2* (*DAG2*) as a key TF regulating rhizome bud dormancy release [29]. In *P. sibiricum*, a transcriptomic study identified 1018 differentially expressed TFs and 519 transcription regulators during stratification and germination [14]. However, the specific functions of these TFs remain unexplored, and whether the regulatory modules characterized in related Polygonatum species are conserved in *P. sibiricum* is unknown, highlighting the need for systematic investigation of TF-mediated regulatory networks in this species.

Despite these advances, the molecular mechanisms underlying seed dormancy release in *P. sibiricum* remain poorly understood. Previous transcriptomic studies have identified numerous differentially expressed genes, including transcription factors and hormone signaling components, modulated during dormancy release [14,20]. However, most of these studies have focused on individual omics layers or static comparisons, and an integrated view of the regulatory networks connecting hormonal signals, transcriptional reprogramming, and metabolic changes during early germination is still lacking. In particular, the extent to which transcription factors coordinate with structural genes to regulate key metabolic pathways remains unexplored.

In this study, we employed integrated transcriptomic and metabolomic approaches to investigate the dynamic changes in gene expression and metabolite profiles during seed dormancy release in *P. sibiricum* at 0, 5, 10, and 15 days after imbibition. We aimed to characterize the physiological changes in hormone and carbohydrate contents, identify key differentially expressed genes and metabolites associated with this process, and elucidate the regulatory roles of starch and sucrose metabolism and plant hormone signal transduction pathways. Furthermore, we constructed a co-expression network linking transcription factors with structural genes in these core pathways to explore potential regulatory hubs. By integrating multi-omics data, we sought to provide a comprehensive framework for understanding the regulatory mechanisms underlying seed dormancy release in *P. sibiricum*, ultimately facilitating the development of effective strategies for improving seed dormancy release and germination in this important medicinal plant.

## 2. Results

### 2.1. Dynamic Changes in Hormone and Carbohydrate Contents During Seed Dormancy Release in P. sibiricum

**Figure 1:**
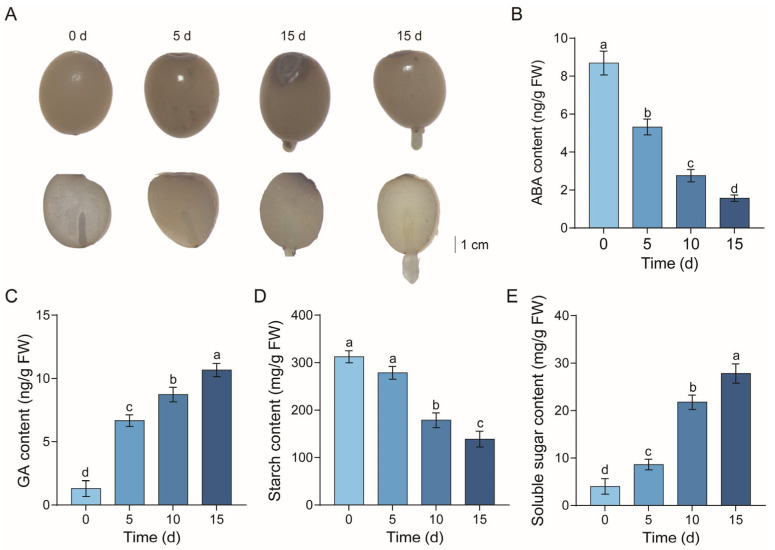
Morphological changes, phytohormonal, and carbohydrate contents dynamics during seed dormancy release in *P. sibiricum*. (**A**) Morphological observations of seeds at 0, 5, 10, and 15 days after sowing under germination conditions (25 °C, 16 h light/8 h dark). (**B**–**E**) Changes in ABA, GA, starch, and soluble sugar contents during seed dormancy release. Data are means ± SD of three biological replicates. Different letters indicate significant differences among time points at *p* value < 0.05 according to Duncan’s multiple range test. FW, fresh weight. Scale bars = 1 cm.

To investigate the physiological mechanisms underlying seed dormancy release in *P. cyrtonema*, the contents of gibberellin (GA), abscisic acid (ABA), starch, and soluble sugar were measured at different time points during the germination process (Figure 1A). As shown in Figure 1B, ABA content remained elevated during the early stage (8.69 mg/g FW at day 0) and gradually declined to 1.57 mg/g FW by day 15, indicating a negative regulatory role of ABA in seed dormancy release. In contrast, GA content increased from 1.30 mg/g FW at day 0 to 10.67 mg/g FW at day 15 (Figure 1B,C). Concurrently, starch content decreased sharply from 312.55 mg/g FW at day 0 to 138.83 mg/g FW at day 15, while soluble sugar content increased from 4.02 mg/g FW to 27.83 mg/g FW over the same period (Figure 1D,E). These results suggest that the transition from dormancy to germination in *P. sibiricum* seeds is accompanied by a shift in hormone balance (increased GA/ABA ratio) and enhanced carbohydrate metabolism, characterized by starch degradation and soluble sugar accumulation, which together provide the necessary energy and osmotic regulation for radicle protrusion and subsequent seedling establishment.

### 2.2. Metabolite Profiling During Different Dormancy Release Stages in P. sibiricum Seeds

**Figure 2:**
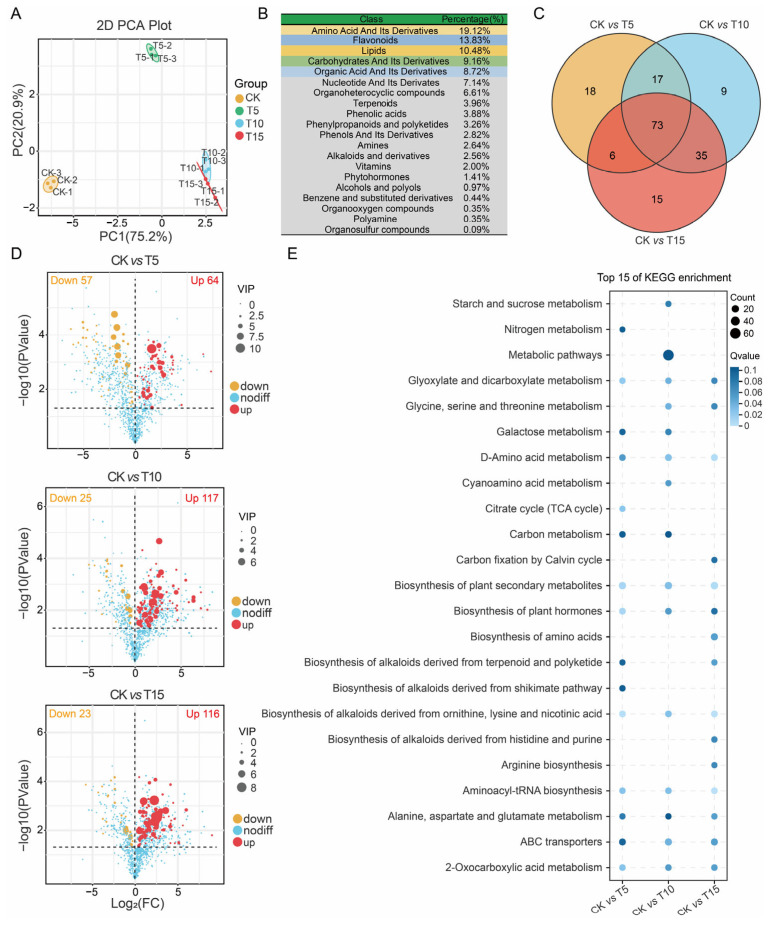
Metabolite analysis on seed dormancy release in *P. sibiricum*. (**A**) Principal component analysis (PCA) of all 12 samples. (**B**) Classification and proportion of metabolites detected in *P. sibiricum* seeds. (**C**) Venn diagram of DEMs in various comparison groups. (**D**) Volcano plots of DEMs in various comparison groups; (**E**) KEGG analysis of DEMs in various comparison groups.

To investigate the dynamic changes in the metabolic profiles of *P. sibiricum* seeds at different stages of dormancy release, LC-MS was employed to profile metabolites from seeds at 0, 5, 10, and 15 days of dormancy release. For monitoring instrumental performance, pooled quality control (QC) samples were analyzed every ten injections throughout the analytical run. As shown in Figure S1, the total ion chromatograms (TICs) of all samples were nearly superimposable, with negligible retention time drift (relative standard deviation < 0.05%). Furthermore, biological replicates from each time point exhibited highly similar TIC profiles, indicating minimal technical variation. These results collectively validated the reliability of the metabolomic dataset. Principal component analysis (PCA) was then conducted to examine the overall metabolic differences between groups and the variations within each group based on the metabolomic data. As shown in Figure 2A, the PCA score plot revealed that the first two principal components (PC1 and PC2) accounted for 75.2% and 20.9% of the total variance, respectively, cumulatively explaining 96.1% of the metabolic variation. In addition, the four stages were clearly separated in the two-dimensional space, indicating substantial metabolic reprogramming during the dormancy release of *P. sibiricum* seeds.

A total of 597 metabolites were detected across these stages, which were classified into 20 categories, including amino acid and its derivatives (19.12%), flavonoids (13.83%), lipids (10.48%), carbohydrates and its derivatives (9.16%), organic acid and its derivatives (8.72%), nucleotide and its derivatives (7.14%), organoheterocyclic compounds (6.61%), terpenoids (3.96%), phenolic acids (3.88%), phenylpropanoids and polyketides (3.26%), phenols and its derivatives (2.82%), amines (2.64%), alkaloids and derivatives (2.56%), vitamins (2.00%), phytohormones (1.41%), alcohols and polyols (0.97%), benzene and substituted derivatives (0.44%), organooxygen compounds (0.35%), polyamines (0.35%), and organosulfur compounds (0.09%), respectively. To further investigate stage-specific metabolic shifts, differentially expressed metabolites (DEMs) were screened based on VIP > 1 and *p* value < 0.05 (Table S1). Compared to the control (CK, 0 d), the number of DEMs showed a progressive increase during germination. Specifically, 118 DEMs (64 upregulated, 54 downregulated) were identified at T5, 134 DEMs (109 upregulated, 25 downregulated) at T10, and 129 DEMs (106 upregulated, 23 downregulated) at T15 (Figure 2D). Venn diagram analysis revealed a core set of 73 DEMs that were commonly altered across all three treatment stages (T5, T10, and T15) relative to CK (Figure 2C). KEGG analysis revealed that DEMs in the various comparison groups were primarily enriched in several metabolic pathways (Figure 2E), such as glyoxylate and dicarboxylate metabolism, D-amino acid metabolism, biosynthesis of plant secondary metabolites, biosynthesis of plant hormones, biosynthesis of alkaloids derived from ornithine, lysine and nicotinic acid, aminoacyl-tRNA biosynthesis, alanine, aspartate, and glutamate metabolism, ABC transporters, and 2-oxocarboxylic acid metabolism.

### 2.3. Transcriptomic Analysis During Different Dormancy Release Stages in P. Sibiricum Seeds

To investigate the dynamic changes in the gene profiles of *P. sibiricum* seeds at different stages of dormancy release, the samples collected at 0, 5, 10, and 15 days were subjected to transcriptomic analysis. Twelve cDNA libraries (CK_1, CK_2, CK_3, T5_1, T5_2, T5_3, T10_1, T10_2, T10_3, T15_1, T15_2, and T15_3) were constructed and sequenced on a PacBio platform. A summary of the RNA-seq data is presented in Table S2. Each library generated more than 36,449,496 raw reads, and after quality filtering, over 4.97 Gb of clean data were retained per sample, with Q30 values ≥ 91.41% and GC contents ranging from 47.30% to 50.36%. In the absence of a reference genome for *P. sibiricum*, the Isoform Sequencing (Iso-Seq) workflow provided by Pacific Biosciences was used to process the raw sequencing reads. A total of 19,493 unigenes were assembled, with a total length of 40,031,929 bp, a maximum length of 7345 bp, and an N50 length of 2254 bp. Functional annotation of the assembled unigenes revealed that 19,006 (97.50%) were annotated in the NCBI non-redundant (NR) database, 16,917 (86.78%) in SwissProt, 18,886 (96.87%) in KEGG, and 13,309 (68.28%) in the eukaryotic complete genomes (KOG) database. Overall, 19,072 genes received functional annotation, while 421 genes remained unannotated. The assembly quality of the transcriptome was determined to be conducive to reliable RNA-seq results.

**Figure 3:**
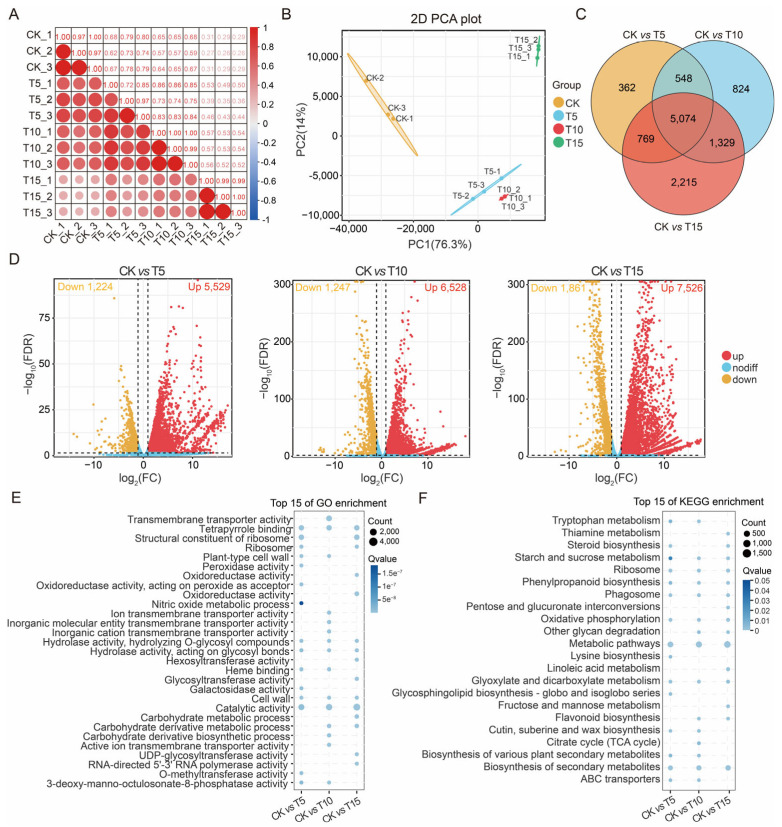
Transcriptomics analysis on seed dormancy release in *P. sibiricum*. (**A**) Sample correlation. (**B**) PCA. (**C**) Venn diagram of DEGs in various comparison groups. (**D**) Volcano plots of DEGs in various comparison groups. (**E**) GO enrichment analysis. (**F**) KEGG enrichment analysis.

As shown in Figure 3A, sample correlation analysis showed high correlations within groups, indicating similar gene expression patterns among replicates at the same stage. PCA revealed that PC1 and PC2 explained 76.30% and 14.00% of the total variance, respectively, effectively grouping the samples into four distinct clusters based on dormancy release stages (Figure 3B). In total, 11 520 expressed genes were quantified across all seed samples, with 6753 (5529 upregulated and 1224 downregulated), 7775 (6528 upregulated and 1247 downregulated), and 9387 (7526 upregulated and 1861 downregulated) genes identified as differentially expressed in the CK vs. T5, CK vs. T10, and CK vs. T15 comparison groups, respectively (Figure 3D). In total, 11,120 DEGs were identified, with 5074 DEGs common to all three comparison groups (Figure 3C). In order to elucidate the functional characteristics of DEGs during different dormancy release stages in *P. sibiricum* seeds, the GO enrichment and KEGG enrichment analyses were performed. The significantly enriched GO terms (top 15) for each comparison group included tetrapyrrole binding, hydrolase activity, cell wall, and catalytic activity (Figure 3E). Furthermore, the KEGG enrichment (top 15) showed that the DEGs in the three comparison groups were mainly involved in pathways related to starch and sucrose metabolism, ribosome, phenylpropanoid biosynthesis, phagosome, oxidative phosphorylation, glyoxylate and dicarboxylate metabolism, and biosynthesis of secondary metabolites (Figure 3F).

### 2.4. Changes in Genes and Metabolites in Starch and Sucrose Metabolism in P. sibiricum Seed Dormancy Release

**Figure 4:**
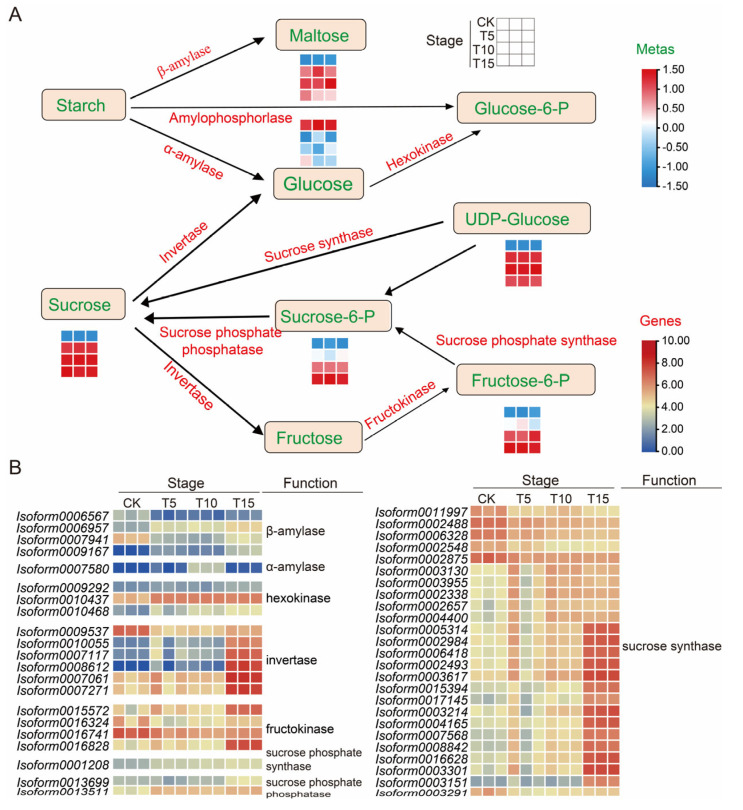
Transcriptomic and metabolomic analysis of starch and sucrose metabolism during seed dormancy release in *P. sibiricum*. (**A**) The model and key components and enzymes of starch and sucrose metabolism. (**B**) Heatmap analysis of DEGs involved in starch and sucrose metabolism.

To investigate the regulatory dynamics of carbohydrate metabolism associated with seed dormancy release in *P. sibiricum*, transcriptomic and metabolomic datasets were integrated with a focus on the starch and sucrose metabolism pathway (KEGG: map00500). The key enzymatic and metabolic intermediates within this pathway are schematically represented in Figure 4A. Metabolomic analysis revealed corresponding changes in the abundance of soluble sugars linked to this pathway. Relative to CK, the levels of sucrose, maltose, UDP-glucose, sucrose-6-P, and fructose-6-P increased progressively across the time course, reaching maximal accumulation at T15. In contrast, glucose content peaked at CK and declined thereafter.

The expression profiles of DEGs encoding key enzymes in starch and sucrose metabolism were visualized as a heatmap, with genes grouped according to their functional categories within the pathway (Figure 4B and Table S3). The heatmap revealed a distinct temporal expression pattern across the four germination stages (0, 5, 10, and 15 days). The majority of these DEGs were upregulated at the later stages of germination (T10 and T15) relative to the control (CK) and the early stage (T5). Specifically, genes annotated as α-amylase (AMY), β-glucosidase (BGLU), and sucrose synthase (SUS) exhibited markedly elevated transcript abundances at T10 and/or T15, whereas genes encoding granule-bound starch synthase (GBSS) showed reduced expression as germination progressed. Biological replicates within each treatment group displayed consistent expression profiles, confirming the reliability of the transcriptomic dataset.

Collectively, the coordinated transcriptional activation of starch and sucrose catabolizing enzymes and the concomitant accumulation of hexose sugars indicate a metabolic shift toward enhanced carbohydrate mobilization during the transition from seed quiescence to active germination in *P. sibiricum*.

### 2.5. Changes in Genes in the Plant Hormone Pathway During Different Dormancy Release Stages in P. sibiricum Seeds

**Figure 5:**
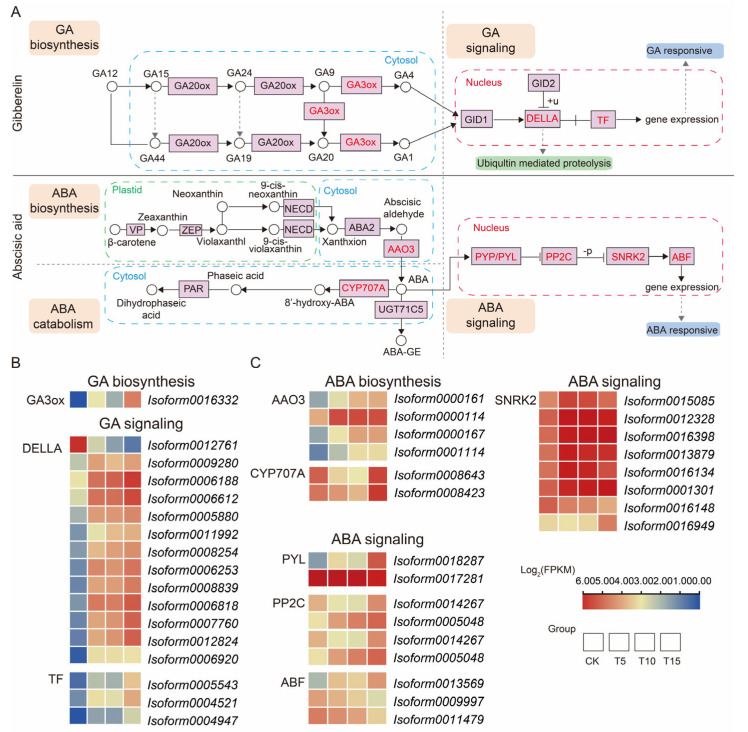
Transcriptomic analysis of the plant hormone pathway during seed dormancy release in *P. sibiricum*. (**A**) The model and key components and enzymes of GA and ABA biosynthesis, catabolism, and signaling pathways. (**B**,**C**) Heatmap analysis of DEGs involved in GA and ABA metabolism. Key enzyme gene abbreviation in the heatmap: GA3ox, gibberellin 3 β-dioxygenase; DELLA, DELLA protein; TF, phytochrome-interacting factor (PIF/PIL); AAO3, abscisic-aldehyde oxidase; CYP707A, (+)-abscisic acid 8’-hydroxylase; PYL, abscisic acid receptor PYR/PYL family; PP2C, protein phosphatase 2C; ABF, ABA responsive element binding factor; SnRK2, serine/threonine-protein kinase SRK2.

During seed dormancy release in *P. sibiricum*, the GA biosynthesis gene GA3ox (*Isoform0016332*) was markedly upregulated. Among DELLA repressors, *Isoform0012761* showed a sustained decrease, whereas most other DELLA transcripts (e.g., *Isoform0006188*, *Isoform0006612*) gradually increased, indicating a complex remodeling of GA signaling. In the ABA pathway, all four AAO3 genes (*Isoform0000161*, *Isoform0000114*, *Isoform0000167*, and *Isoform0001114*) were continuously upregulated, while the catabolic CYP707A genes (*Isoform0008643* and *Isoform0008423*) were markedly suppressed at early and middle stages but rebounded to higher levels at the late stage (Figure 5B and Table S4). For ABA signaling, one PYL receptor gene (*Isoform0018287*) was strongly induced at the late stage, whereas another PYL (*Isoform0017281*) declined. A PP2C negative regulator (*Isoform0005048*) increased steadily, whereas positive regulators SnRK2s (e.g., *Isoform0016398*) peaked at the late stage. Downstream ABF transcription factors showed divergent trends, with *Isoform0013569* continuously upregulated and *Isoform0009997* downregulated (Figure 5C and Table S4). Collectively, these data indicate that dormancy release in *P. sibiricum* seeds involves a finely tuned antagonistic interplay between the GA and ABA pathways, characterized by stage-specific reprogramming of both hormone metabolism and signal transduction genes.

### 2.6. TFs Analysis in RNA-seq Data

**Figure 6:**
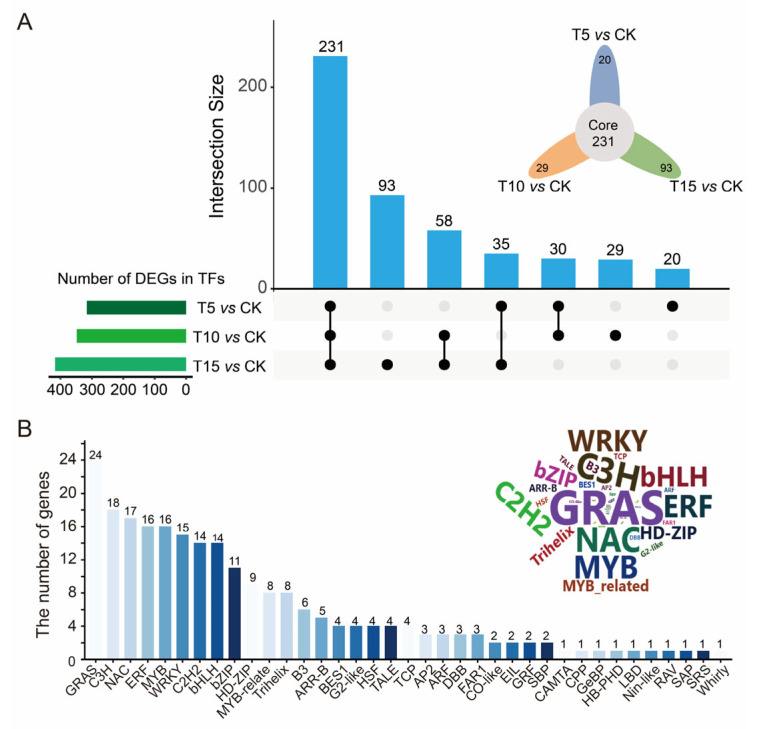
Analysis of transcription factors among DEGs during different dormancy release stages in *P. sibiricum* seeds. (**A**) Upset plots of differential TF expression in three different comparison groups. (**B**) Statistics of differentially expressed transcription factor family numbers in the core group.

Based on the transcriptome data, a global survey of differentially expressed transcription factors (DETFs) during seed dormancy release in *P. sibiricum* (0, 5, 10, and 15 d) was performed. In total, 316, 412, and 479 DETFs were identified at T5 vs. CK, T10 vs. CK, and T15 vs. CK, respectively. Among these, 231 TFs were differentially expressed in all comparison groups (Figure 6A). In terms of family distribution, the GRAS family was the largest, comprising 24 members, followed by the C3H (18 members), NAC (17 members), ERF and MYB (16 members), WRKY (15 members), C2H2 and bHLH (14 members), bZIP (11 members), HD-ZIP (9 members), MYB_related and Trihelix (8 members), B3 (6 members), ARR-B (5 members), BES1, G2-like, HSF, TALE, and TCP (4 members), AP2, ARF, DBB, and FAR1 (3 members), CO-like, EIL, GRF, and SBP (2 members); CAMTA, CPP, GeBP, HB-PHD, LBD, Nin-like, RAV, SAP, SRS, and Whirly (1 member) (Figure 6B). The core TF network during seed dormancy release in *P. sibiricum* is characterized by widespread activation of families such as AP2, ARR-B, BES1, bHLH, bZIP, C3H, ERF, GRAS (except DELLA), HD-ZIP, and MYB, alongside repression of NAC, WRKY, HSF, and specific members of ARF, B3, C2H2, and TCP (Table S5). This coordinated transcriptional reprogramming likely underpins the transition from dormancy to active germination.

### 2.7. Combined Transcriptome and Metabolome Analysis

**Figure 7:**
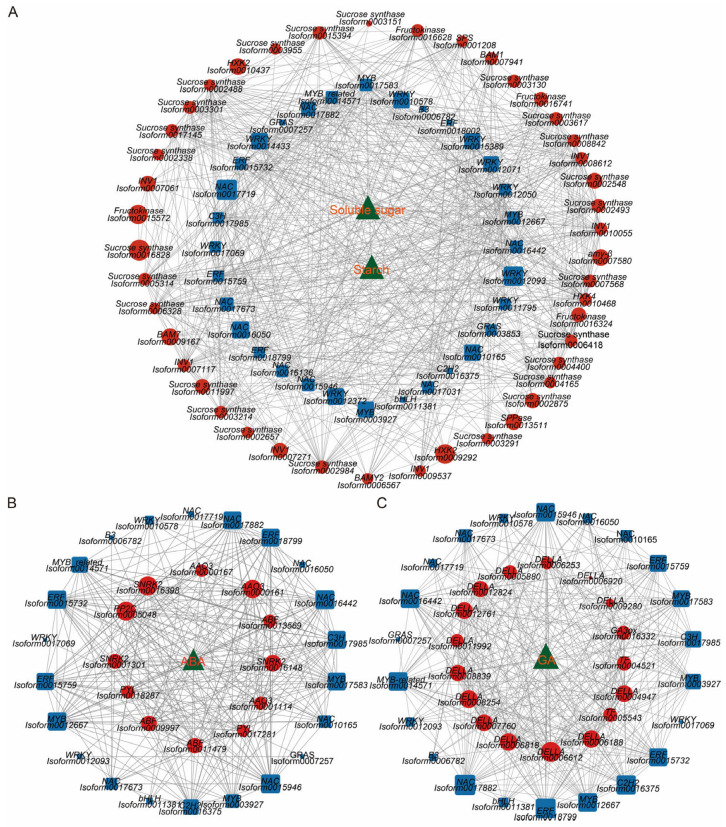
Co-expression network connecting structural genes in starch and sucrose metabolism and the plant hormone pathway with transcription factors (TFs). (**A**) Starch and sucrose metabolism. (**B**) ABA-related pathway. (**C**) GA-related pathway. Dark green triangles denote metabolites; red circles denote DETFs; and round rectangles denote key enzymes in core pathways. The size of the node shape indicates the degree of connectivity, with a larger size representing a higher degree, and vice versa.

To investigate the key TFs involved in starch and sucrose metabolism and plant hormone signaling pathways, we conducted further analyses based on a co-expression network connecting structural genes with TFs, which was constructed across all comparison groups. The correlation network was constructed based on Spearman correlation analysis. Nodes with an absolute coefficient (|r|) greater than 0.8 and a *p* value less than 0.05 were connected to visualize significant associations [30]. A total of 31 TFs were associated with starch and sucrose metabolism, 21 with the ABA signaling pathway, and 22 with the GA signaling pathway; among these, 19 TFs were common to all three groups (Figure 7 and Table S6). The expression profiles of these 19 shared TFs across four time points (CK, T5, T10, T15) revealed distinct temporal patterns. The majority of these TFs exhibited significant downregulation after treatment, with the most dramatic decrease observed for a MYB family member. Notably, only one TF, belonging to the C2H2 family, showed sustained and significant upregulation throughout the time course, suggesting a potential positive regulatory role in the response. Another C3H family member maintained relatively higher levels at the early treatment stages but gradually decreased afterward, yet remained lower than the control. The NAC families, which contained the largest number of shared TFs, were all significantly downregulated upon treatment, whereas the MYB family also exhibited strong and persistent repression. Overall, the coordinated downregulation of most shared TFs, together with the specific upregulation of a C2H2 TF, highlights a potential transcriptional reprogramming event that may underpin the crosstalk between carbon metabolism and hormone signaling under the experimental conditions.

### 2.8. RT-qPCR Validation of RNA-seq Data

**Figure 8:**
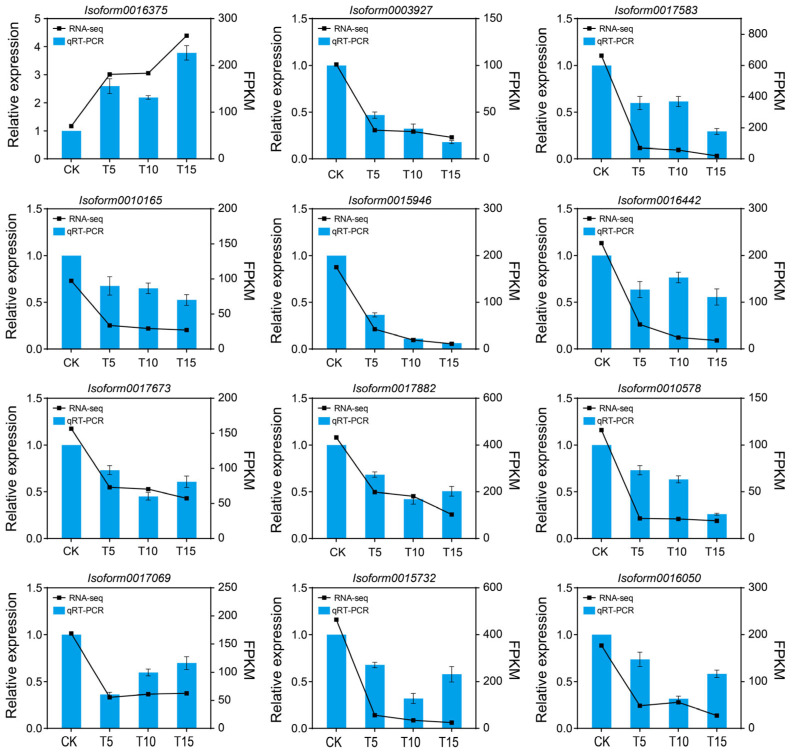
RT-qPCR validation and RNA-seq data expression pattern analysis of DETFs.

Quantitative real-time PCR (RT-qPCR) was employed to validate the expression patterns of twelve transcription factor genes that were identified to be associated with key metabolic pathways. The results showed that the expression trends observed by RT-qPCR were generally consistent with the FPKM values derived from the RNA-seq data (Figure 8). As the germination time of *P. sibiricum* seeds increased, the expression levels of most transcription factors exhibited a declining trend, with the exception of *Isoform0016375*, which showed increased expression over time. These findings further confirm the reliability of the transcriptome sequencing data and suggest that these transcription factor genes may be involved in the release of seed dormancy in *P. sibiricum*.

## 3. Discussion

### 3.1. Global Analysis of Dynamic Changes During P. sibiricum Seed Dormancy Release by Transcriptomic and Metabolic Profiling

Integrated transcriptomic and metabolomic analyses across four time points (0, 5, 10, and 15 days after imbibition) revealed progressive and substantial molecular reprogramming during dormancy release. PCA of both datasets clearly separated the four stages, with metabolomic PC1 and PC2 cumulatively explaining 96.1% of the variance, indicating that the observed changes are highly robust and primarily driven by the dormancy-release process. Previous studies in congeneric Polygonatum species have typically adopted coarse temporal resolutions. For instance, in *P. cyrtonema*, analyses were performed by comparing only two stages (ungminated versus germinated seeds) [19], or at most three stages: dormancy, germination, and seedling emergence [18]. Similarly, transcriptomic profiling of *P. sibiricum* during dormancy release has been conducted at widely spaced intervals (0, 30, 60, 90, and 105 days), with the earliest sampling point at 30 days after treatment initiation, a time point at which seeds had already been dormant for one month [14]. These study designs, while providing valuable snapshots of the initial dormant state and the final germinated state, largely overlooked the dynamic transcriptional and metabolic events occurring during the critical early transition period from imbibition to dormancy release. In contrast, our study employed a finely resolved time course (0, 5, 10, and 15 days after imbibition), capturing the earliest molecular changes that precede visible germination. This high-resolution temporal design enabled the identification of stage-specific regulatory programs that would otherwise be masked in coarse comparative designs.

The number of DEMs and DEGs increased progressively over time, from 118 DEMs and 6753 DEGs at T5 to 129 DEMs and 9387 DEGs at T15, indicating that dormancy release is a gradual process involving sequential activation of transcriptional and metabolic programs. Notably, the number of DEGs identified in our study (up to 9387 at T15) is close to the 11,817 unigenes reported in *P. cyrtonema* between germinated and ungerminated seeds [31], suggesting that the early phase of dormancy release in *P. sibiricum* involves transcriptional reprogramming comparable in scale to the entire germination process in the related species.

Metabolite profiling revealed that amino acids and their derivatives (19.12%) and carbohydrates and their derivatives (9.16%) were among the most abundant classes, consistent with the central roles of nitrogen mobilization and energy metabolism during germination. Flavonoids (13.83%) were also highly represented, potentially reflecting species-specific accumulation of phenolic compounds related to the medicinal properties of *P. sibiricum*. KEGG enrichment of DEMs highlighted glyoxylate and dicarboxylate metabolism, D-amino acid metabolism, and biosynthesis of plant secondary metabolites, while transcriptomic enrichment identified starch and sucrose metabolism, ribosome, phenylpropanoid biosynthesis, and oxidative phosphorylation as key pathways. The co-enrichment of starch and sucrose metabolism at both levels underscores the central role of carbohydrate mobilization in providing energy for germination [17,19], while the enrichment of phenylpropanoid biosynthesis suggests active cell wall remodeling to facilitate radicle emergence, which has been previously documented in *P. sibiricum* through physiological studies of cell wall-degrading enzymes [32]. The strong concordance between transcriptomic and metabolomic datasets validates the reliability of our multi-omics approach and provides a solid foundation for dissecting the regulatory networks governing dormancy release in *P. sibiricum*.

### 3.2. Plant Hormones Signals and Starch and Sucrose Metabolism Play Important Roles in P. sibiricum Seed Dormancy Release

The antagonistic balance between ABA, which promotes and maintains dormancy, and GAs, which promote germination, is widely recognized as the central hormonal switch controlling the dormancy-to-germination transition [16,33,34]. Our physiological measurements demonstrated that ABA content decreased progressively from 8.69 mg/g FW at day 0 to 1.57 mg/g FW at day 15, whereas GA content increased from 1.30 to 10.67 mg/g FW over the same period, resulting in a markedly elevated GA/ABA ratio (Figure 1B,C). This shift is consistent with previous studies on quinoa seed germination, where ABA content decreased significantly upon imbibition and continued to decline during germination, accompanied by upregulation of GA biosynthetic genes [35]. Similarly, in *P. cyrtonema*, ABA content showed a significant decline during germination, and plant hormone signal transduction was identified as a key pathway enriched with differentially expressed genes during seed germination [19,31].

At the transcriptional level, our data revealed that the GA biosynthesis gene *GA3ox* (*Isoform0016332*) was markedly upregulated during dormancy release, whereas the *DELLA* repressor (*Isoform0012761*) exhibited a sustained decrease (Figure 5B), consistent with the established model that GA promotes DELLA degradation via the 26S proteasome pathway to relieve repression of germination-associated genes [16]. Notably, most other *DELLA* transcripts (e.g., *Isoform0006188* and *Isoform0006612*) gradually increased, indicating a complex, isoform-specific remodeling of GA signaling rather than a simple global suppression of all DELLA members. This pattern is consistent with observations in *P. cyrtonema*, where the GA/ABA ratio increased markedly during germination, reflecting the antagonistic interplay between these two hormones [35].

In the ABA pathway, all four *AAO3* genes (*Isoform0000161*, *Isoform0000114*, *Isoform0000167*, and *Isoform0001114*) were continuously upregulated, while the catabolic *CYP707A* genes (*Isoform0008643* and *Isoform0008423*) were markedly suppressed at early and middle stages but rebounded at the late stage (Figure 5B). This biphasic expression pattern suggests that ABA biosynthesis remains active during dormancy release, with catabolism playing a more prominent role at later stages. The temporal dynamics of CYP707A expression observed here differ from findings in some other species, suggesting species-specific regulatory strategies. In *Arabidopsis*, *CYP707A* genes play differential roles in regulating seed germination, with *CYP707A1* and *CYP707A2* being indispensable for proper control of seed dormancy and germination [36]. For ABA signaling, the receptor gene *PYL* (*Isoform0018287*) was strongly induced at the late stage, whereas another *PYL* (*Isoform0017281*) declined. The negative regulator *PP2C* (*Isoform0005048*) increased steadily, while positive regulators *SnRK2s* (e.g., Isoform0016398) peaked at the late stage (Figure 5C). This dynamic interplay between receptors, phosphatases, and kinases indicates that ABA signaling is not simply turned off but rather undergoes a stage-specific reconfiguration during dormancy release. In *P. cyrtonema*, proteomic analysis revealed that ABA-related proteins such as PYL4 and PP2C decreased during germination [34], suggesting that attenuation of ABA signaling is a conserved feature of dormancy release in Polygonatum species.

Concurrently with hormonal changes, starch and sucrose metabolism emerged as a central pathway during dormancy release. Starch content decreased sharply from 312.55 to 138.83 mg/g FW, while soluble sugar content increased from 4.02 to 27.83 mg/g FW over the same period (Figure 1D,E). Metabolomic analysis revealed corresponding changes in the abundance of soluble sugars linked to this pathway. Relative to CK, the levels of sucrose, maltose, UDP-glucose, sucrose-6-P, and fructose-6-P increased progressively across the time course, reaching maximal accumulation at T15, whereas glucose content peaked at CK and declined thereafter (Figure 4B). This pattern suggests that stored starch is progressively hydrolyzed to soluble sugars, which are then utilized for energy production via glycolysis and the TCA cycle to support radicle emergence. At the transcriptional level, genes encoding α-amylase (AMY), β-glucosidase (BGLU), and sucrose synthase (SUS) were markedly upregulated at T10 and/or T15, whereas granule-bound starch synthase (GBSS) showed reduced expression as germination progressed (Figure 4B). This transcriptional shift from starch synthesis to degradation is consistent with findings in *P. cyrtonema*, where starch and sucrose metabolism were among the most significantly enriched pathways, with 59 DEGs detected (41 upregulated and 18 downregulated) [19,31]. The *α-amylase* gene and *maltase-glucoamylase* (MGAM) enzyme genes were significantly upregulated during seed germination, and α-amylase activity was highest in germinated seeds [19]. Similarly, in *P. cyrtonema* seeds treated with GA_4+7_, soluble sugar content and α-amylase activity were significantly increased, while pectin content decreased, indicating enhanced carbohydrate mobilization and cell wall degradation [37]. In *P. sibiricum*, studies on endosperm weakening revealed that β-mannanase, β-galactosidase, and polygalacturonase play important positive roles in endosperm weakening [32], consistent with the upregulation of hydrolase-related genes observed in our transcriptomic data. The accumulation of UDP-glucose may also serve as a precursor for cell wall biosynthesis, linking carbohydrate metabolism to the structural requirements of germination. Furthermore, during *P. sibiricum* seed stratification, starch and cellulose were identified as the main metabolic substances, with jasmonates, auxins, and ABA jointly maintaining dormancy, while GA_7_ and ACC promote dormancy release and germination [38].

### 3.3. Transcription Factor Networks Integrate Hormonal and Metabolic Signals

Transcription factors serve as key nodes integrating hormonal signals and metabolic reprogramming during seed germination [16]. Our global survey identified 316, 412, and 479 differentially expressed transcription factors at T5, T10, and T15, respectively, indicating progressively intensifying transcriptional reprogramming as dormancy release proceeds. In terms of family distribution, the GRAS family was the largest, comprising 24 members, followed by C3H (18), NAC (17), ERF and MYB (16), WRKY (15), C2H2 and bHLH (14), bZIP (11), HD-ZIP (9), MYB_related and Trihelix (8), and B3 (6) (Figure 6B). The predominance of the GRAS family, which includes DELLA proteins, underscores the central role of GA signaling in this process. DELLA proteins are well-established as master repressors of GA responses, acting as key negative regulators that restrain growth and germination through the 26S proteasome degradation pathway [39,40,41]. In soybean, GRAS genes exhibit diverse expression patterns during seed germination [42]. The NAC family (17) was the third largest and predominantly downregulated. NAC transcription factors are known to regulate secondary cell wall formation and seed germination [43,44]. In *Arabidopsis*, *NAC25* and *NAC1L* have been shown to regulate endosperm cell expansion and cell wall modification during germination [45]. However, the downregulation of most NAC genes in our data contrasts with the upregulation of their counterparts in other species, suggesting that different NAC members may play divergent roles across species or stages. The MYB and ERF families (16 members each) showed mixed expression patterns, with some members upregulated and others downregulated, reflecting their involvement in diverse signaling pathways. In *Arabidopsis*, *MYB96* mediates ABA signaling to inhibit germination [23], whereas *MYB30* functions downstream of ABA receptors to promote NO-induced dormancy breaking [46]. Similarly, ERF transcription factors exhibit dual roles in germination: *ERF1* promotes germination by enhancing mitochondrial function and modulating ROS homeostasis [47], while *ZmEREB92* acts as a negative regulator by suppressing ethylene signaling and starch mobilization [48]. The WRKY family (15 members) was predominantly downregulated, consistent with its roles in ABA signaling and stress responses during seed dormancy. *WRKY25* promotes primary seed dormancy through the DOG1 and ABA signaling pathways [27], whereas *OsWRKY71* functions as a master regulator of germination in rice by modulating GA and ABA signaling dynamics [49], and *TgWRKY24* promotes germination by transcriptionally activating ABA catabolism [50].

A particularly interesting finding emerged from the co-expression network analysis connecting structural genes with TFs. A total of 31 TFs were associated with starch and sucrose metabolism, 21 with the ABA signaling pathway, and 22 with the GA signaling pathway; among these, 19 TFs were common to all three groups (Figure 7 and Table S6). The expression profiles of these 19 shared TFs across four time points (CK, T5, T10, T15) revealed distinct temporal patterns. The majority of these TFs exhibited significant downregulation after treatment, with the most dramatic decrease observed for a MYB family member. Notably, only one TF, belonging to the C2H2 family, showed sustained and significant upregulation throughout the time course, suggesting a potential positive regulatory role in the response. The NAC family, which contained the largest number of shared TFs, was significantly downregulated upon treatment, whereas the MYB family also exhibited strong and persistent repression. Overall, the coordinated downregulation of most shared TFs, together with the specific upregulation of a C2H2 TF, highlights a potential transcriptional reprogramming event that may underpin the crosstalk between carbon metabolism and hormone signaling during dormancy release.

### 3.4. Coordinated Regulation of the Dormancy-to-Germination Transition

Taken together, our results support an integrated model for seed dormancy release in *P. sibiricum* in which hormonal signals, metabolic reprogramming, and transcription factor networks are coordinately regulated to drive the transition from dormancy to germination. In this model, the onset of germination triggers a progressive shift in the ABA/GA balance, driven by declining ABA content and increasing GA biosynthesis. The elevated GA/ABA ratio promotes DELLA degradation, relieving repression of downstream transcription factors such as NACs and MYBs. These transcription factors, in turn, activate genes involved in starch hydrolysis (e.g., AMY, BGLU), cell wall remodeling (e.g., endoglucanase, XTHs), and energy metabolism, providing the resources necessary for radicle protrusion and subsequent germination.

The coordinated downregulation of most shared transcription factors in the co-expression network, together with the specific upregulation of a C2H2 member, suggests that both release from repression and targeted activation contribute to the transcriptional reprogramming underlying dormancy release. This dual regulatory mechanism may ensure the precise temporal control of germination-related genes, preventing premature germination while allowing a rapid response to favorable conditions. The progressive increase in DEGs and DEMs over time further supports the notion that dormancy release is not a single switch but rather a multi-step process involving sequential activation of distinct regulatory modules.

Our findings are largely consistent with previous studies on seed germination in related Polygonatum species. In *P. cyrtonema*, transcriptomic and metabolomic analyses revealed that DEGs were significantly enriched in the flavonoid synthesis and phenylpropanoid metabolism pathways, with flavonoids significantly accumulated and hydroxycinnamoyl derivatives significantly decreased in germinated seeds. The seed coat of *P. cyrtonema* seeds is constituted by a layer of lignified cells, and lignin represents a physical barrier to seed germination. Our GO enrichment analysis identified “cell wall” and “hydrolase activity” as significantly enriched terms (Figure 3E), and KEGG enrichment highlighted phenylpropanoid biosynthesis (Figure 3F), further supporting the involvement of cell wall-related processes during dormancy release in *P. sibiricum*. In *P. sibiricum*, studies on endosperm weakening during seed germination revealed that β-mannanase, β-galactosidase, and polygalacturonase play important positive roles in endosperm weakening. This is consistent with the observation that cell wall degradation in *P. sibiricum* seeds is mediated by multiple hydrolases acting cooperatively on different cell wall components (cellulose, hemicellulose, and pectin) to achieve endosperm weakening.

## 4. Materials and Methods

### 4.1. Plant Materials and Treatment

*P. sibiricum* seeds (cultivar *DJ1*) that were dry, plump, uniform in size, and free from mold and deterioration were selected as the experimental material. This cultivar exhibits relatively weak seed dormancy compared with other accessions, as determined by our preliminary screening. The selected seeds were sterilized with 3% H_2_O_2_ for 15 min and then thoroughly rinsed three times with distilled water. Subsequently, the seeds were soaked in sterile water for 24 h in an incubator set at 25 ± 1 °C, after which they were thoroughly rinsed three times with distilled water.

The treated seeds were then placed in germination boxes containing sterilized sand or vermiculite. Each box contained 50 seeds. The boxes were subsequently placed in an illuminated incubator. The incubation conditions were set as follows: temperature at 25 °C, light intensity at 2500 lx, photoperiod of 12 h light/12 h dark, and ambient humidity at 70%. Each treatment was performed in triplicate. To investigate the regulatory mechanisms of seed dormancy release, intact seeds were collected at 0, 5, 10, and 15 d after treatment initiation. At each time point, 50 seeds were randomly sampled from each of three independent replicate boxes, carefully removed from the substrate, rinsed with distilled water, and visually examined for morphological changes before being immediately frozen in liquid nitrogen for subsequent physiological and omics analyses.

### 4.2. Quantification of Hormones and Carbohydrates

ABA and GA Contents (ELISA): A 0.5 g frozen sample was ground in an ice bath with 5 mL of 80% extraction buffer containing 1 mmol/L butylated hydroxytoluene (BHT). The homogenate was kept at 4 °C in the dark for 12 h and then centrifuged at 10,000 rpm for 20 min. The supernatant was collected and added to microplate wells pre-coated with specific monoclonal antibodies against the hormones. After blocking, the solution was successively mixed with primary antibody and horseradish peroxidase (HRP)-conjugated secondary antibody. Color development was then carried out using 3,3’,5,5’-tetramethylbenzidine (TMB) substrate, and the reaction was stopped with the addition of sulfuric acid. Absorbance was finally measured at 450 nm with a reference wavelength of 630 nm.

Soluble sugar and starch contents were determined using the anthrone–sulfuric acid colorimetric method. Briefly, accurately weighed dried and sieved plant samples were extracted with 80% ethanol in a water bath to obtain soluble sugar extracts. After centrifugation, the supernatants were combined and adjusted to a constant volume for the determination of soluble sugar, while the residual precipitates were retained for subsequent starch hydrolysis. For soluble sugar quantification, the extract was mixed with freshly prepared anthrone–sulfuric acid reagent, heated in a boiling water bath, and cooled to room temperature. The absorbance was measured at 620 nm using a spectrophotometer, and the soluble sugar content was calculated according to the standard curve established with glucose standard solutions. For starch content determination, the ethanol residues remaining after soluble sugar extraction were treated with distilled water to remove residual ethanol, followed by acid hydrolysis with 2 mol/L hydrochloric acid in a boiling water bath to completely decompose starch into glucose. After cooling, the hydrolysate was neutralized with 2 mol/L sodium hydroxide solution to a neutral pH, and then fixed to a constant volume and centrifuged. The supernatant was collected and reacted with anthrone–sulfuric acid reagent under the same heating and colorimetric conditions as soluble sugar determination. The starch content was calculated based on the glucose concentration obtained from the standard curve with a conversion coefficient of 0.9 for glucose to starch. All experiments were performed in triplicate.

### 4.3. Metabolomic Analysis

A total of 0.1 g of seeds was individually ground in liquid nitrogen, and the homogenate was resuspended in 500 μL of pre-chilled 80% methanol. After vortexing, the mixture was incubated on ice for 5 min, and then centrifuged at 15,000 rpm for 20 min at 4 °C. An aliquot of the supernatant was diluted with LC–MS grade water to a final methanol concentration of 53%, followed by a second centrifugation at 15 000× *g* for 20 min at 4 °C. The resulting supernatant was collected for LC-MS (HPLC, Exion LC, SCIEX; MS, QTRAP 6500 + SCIEX) analysis. Samples were injected onto a chromatographic column (150 × 2.1 mm, 2.5 µm) using a 17 min linear gradient at a flow rate of 0.4 mL/min. The eluents were eluent A (0.1% FA in water) and eluent B (0.1% FA in acetonitrile). The solvent gradient was set as follows: 2% B, 2 min; 2–100% B, 15.0 min; 100% B, 17.0 min; 100–2% B, 17.1 min; 2% B, 20 min. The QTRAP 6500+ mass spectrometer was operated in both positive and negative polarity modes. The parameters were set as follows: curtain gas at 35 psi, collision gas set to medium, ion spray voltage at 5500 V for positive mode and −4500 V for negative mode, temperature at 550 °C, and ion source gases 1 and 2 both at 60 psi.

Metabolite detection was performed using multiple reaction monitoring (MRM) based on an in-house database. For metabolite quantification, the peak area of each MRM transition (Q3) was used, while metabolite identification was achieved by matching Q1, Q3, retention time (RT), declustering potential (DP), and collision energy (CE) against the database. Raw data files generated by HPLC-MS/MS were processed using SCIEX OS software (Version 1.4) for peak integration and correction. The main parameters were set as follows: minimum peak height of 500, signal-to-noise ratio ≥ 5, and Gaussian smoothing width of 1. The peak area of each metabolite represented its relative content.

### 4.4. Full-Length Transcriptome Sequencing and Analysis

Total RNA was extracted from the samples using TRIzol Reagent (Thermo Fisher Scientific, Waltham, MA, USA) in accordance with the manufacturer’s protocol. RNA-seq libraries were constructed and sequenced on the PacBio Sequel III platform by Gene Denovo Biotechnology Co. (Guangzhou, China). The raw sequencing data were processed using the PacBio IsoSeq pipeline. Circular consensus sequences (CCS, HiFi reads) were extracted, and full-length non-chimeric (FLNC) reads were identified on the basis of the presence of 5’ and 3’ primers and poly-A tails. FLNC reads were clustered using minimap2 to generate consensus isoforms, which were then polished with the Quiver algorithm to obtain high-quality transcripts (accuracy ≥ 0.99). The prediction of coding sequences (CDSs) was conducted utilizing the ANGEL software. For the purpose of functional annotation, the isoforms were aligned against the Nr (https://ftp.ncbi.nlm.nih.gov/blast/db, accessed on 31 luly 2026), Swiss-Prot (https://www.expasy.org/resources/uniprotkb-swiss-prot, accessed on 31 luly 2026), KEGG (https://www.genome.jp/kegg, accessed on 31 luly 2026), and COG/KOG (https://www.genome.jp/kegg, accessed on 31 luly 2026) databases using BLASTx (E-value < 1e^−5^). The assembled isoforms from the PacBio sequencing were used as the reference transcriptome. Subsequently, short-read Illumina sequencing data were aligned to these reference isoforms, and transcript abundances were quantified using RSEM software. After normalization for sequencing depth and transcript length, gene expression levels were calculated as fragments per kilobase of transcript per million mapped reads (FPKM). The differential expression gene (DEG) analysis was performed using the DESeq2 software with thresholds set at a |log_2_ (fold change, FC)| ≥ 1 and a false discovery rate (FDR) < 0.05. Functional enrichment of the identified DEGs was evaluated using a hypergeometric test against the KEGG pathway and Gene Ontology (GO) databases.

### 4.5. Verification of RT-qPCR

Key DEGs identified from transcriptome analysis were selected for RT-qPCR validation. Each sample was analyzed with three biological replicates. GAPDH was used for normalization [51]. The relative expression levels were calculated according to the 2^−ΔΔCt^ method [52]. The primers used are listed in Table S7.

### 4.6. Statistical Analysis

The statistical analysis of the data was conducted using SPSS (v26.0). Normality and homogeneity of variances were first verified using the Shapiro–Wilk test and Levene’s test, respectively. As the assumptions for parametric tests were satisfied, a one-way analysis of variance (ANOVA) was employed to compare the means across different treatment groups. Subsequently, Duncan’s multiple range test was applied as a post hoc measure for pairwise comparisons. The statistical significance threshold was set at *p* value < 0.05. Graphs were constructed using GraphPad Prism 10.

## 5. Conclusions

In this study, integrated transcriptomic and metabolomic analyses revealed that seed dormancy release in *Polygonatum sibiricum* is governed by coordinated hormonal, metabolic, and transcriptional reprogramming. The shift in ABA/GA balance, driven by declining ABA and increasing GA, along with enhanced starch degradation and soluble sugar accumulation, provides the physiological foundation for germination. Key events include upregulation of GA3ox, sustained downregulation of the DELLA repressor Isoform0012761, and stage-specific remodeling of ABA signaling components. Starch and sucrose metabolism emerged as a central pathway, with hydrolytic enzymes activated to mobilize energy reserves. Transcriptionally, 316, 412, and 479 differentially expressed transcription factors were identified at T5, T10, and T15, with the GRAS family being the largest. Co-expression network analysis further identified 19 transcription factors integrating hormone and metabolic signaling, with most showing coordinated downregulation and one C2H2 member exhibiting sustained upregulation. Collectively, our findings provide a comprehensive framework for understanding the regulatory network underlying dormancy release in *P. sibiricum* and identify candidate genes and transcription factors as potential targets for improving seed germination in this important medicinal plant.

## Data Availability

The original contributions presented in this study are included in the article/Supplementary Material. Further inquiries can be directed to the corresponding author.

## Supplementary Materials

The following supporting information can be downloaded at https://www.mdpi.com/article/10.3390/ijms27157032/s1.
